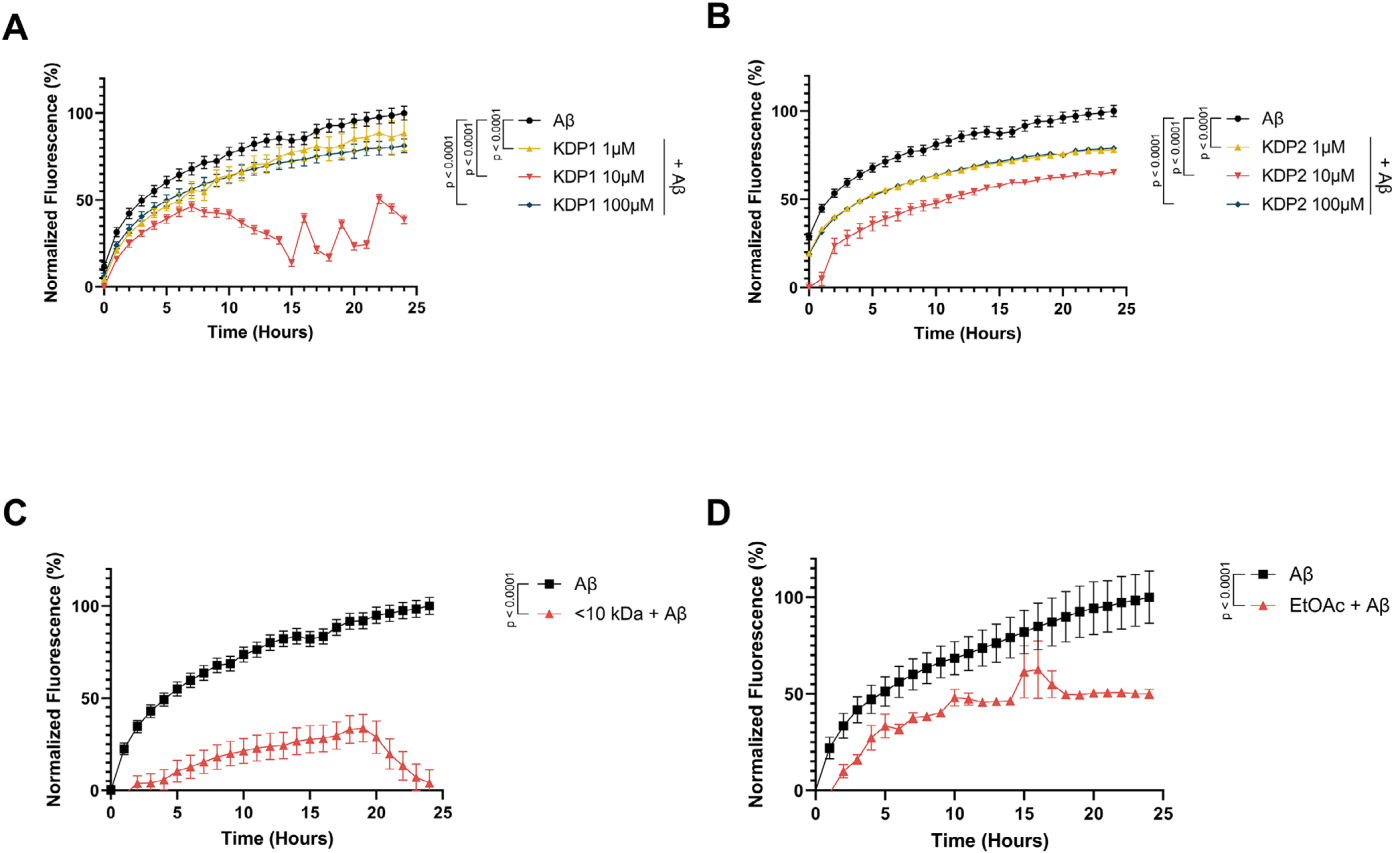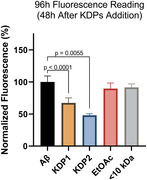# A Follow‐Up Investigation: In Vitro Effects of Kefir‐Derived Biomolecules on β‐Amyloid Aggregation

**DOI:** 10.1002/alz70859_103543

**Published:** 2025-12-25

**Authors:** Serena Mares Malta, Lucas Matos Martins Bernardes, Matheus Henrique Silva, Ana Carolina Costa Santos, Letícia Leandro Batista, Tamiris Sabrina Rodrigues, Fernanda Araújo do Prado Mascarenhas, Renata Graciele Zanon, Foued Salmen Espindola, Ana Paula Mendes‐Silva, Carlos Ueira‐Vieira

**Affiliations:** ^1^ University of Saskatchewan, Saskatoon, SK Canada; ^2^ Universidade Federal de Uberlândia, Uberlândia, Minas Gerais Brazil; ^3^ Johannes Gutenberg‐Universität Mainz, Mainz, Rheinland Pfalz Germany

## Abstract

**Background:**

Kefir is a probiotic‐rich fermented milk beverage composed of a symbiotic consortium of bacteria and yeasts. Emerging evidence has shown its neuroprotective potential, including that of its derived metabolites and fractions, in mitigating β‐amyloid (Aβ42)‐induced neurotoxicity in cultured neuronal cells and neurodegeneration in *Drosophila melanogaster* models for Alzheimer’s disease (AD). Building on these findings, we explored the *in vitro* effects of kefir‐derived fractions and synthetic peptides on Aβ42 aggregation and disaggregation.

**Method:**

Two kefir fractions, Ethyl Acetate (EtOAc) and <10kDa, and two kefir‐derived peptides (KDPs) identified in our prior research were tested. For the preventive assay, Aβ42 (10 µM) was co‐incubated with kefir fractions (0.25 mg/mL) or KDPs (1, 10 and 100 µM) for 24 hours, with fluorescence readings (Thioflavin T) taken hourly. For the treatment assay, Aβ42 was incubated alone for 48 hours to induce aggregation, followed by treatment with fractions or KDPs, with fluorescence readings taken after an additional 48‐hour incubation. All experiments were performed in 96‐well plates, with samples in quintuplicate. Statistical analysis was conducted using one‐way ANOVA.

**Result:**

Fluorescence intensity measurements revealed that, in the preventive assay, all treatments significantly reduced Aβ42 aggregation compared to the untreated control (p<0.0001). In the treatment assay, significant disruption of Aβ42 aggregation was observed with KDP‐1 (p=0.0055) and KDP‐2 (p<0.0001).

**Conclusion:**

This study highlights the potential ability of kefir fractions and synthetic peptides to prevent and disrupt Aβ42 aggregation *in vitro*, supporting their therapeutic promise in neurodegenerative disorders. Further studies should explore their mechanisms of action and efficacy *in vivo*.